# The Impact of Certified Peer Specialists as Teachers to Families

**DOI:** 10.1007/s10597-026-01622-9

**Published:** 2026-04-16

**Authors:** Nicole Cerundolo, Emily Carol, Adrienne Gerken, Kirsten Bolton, Rahel Bosson, Ruth Firmin, Jamie Fischer, Zachary Millman, Nathaniel Van Kirk, Stephen Fedele

**Affiliations:** 1 McLean Hospital, Belmont, USA; 2 Northeastern University, Boston, USA; 3 Harvard Medical School, Boston, USA; 4 Thomas Jefferson University Hospital, Philadelphia, USA; 5 Sidney Kimmel Medical College, Philadelphia, USA; 6 School of Psychology and Human Services, Gordon College, Wenham, USA

## Abstract

Certified peer specialists (CPSs) are providers with lived experience of mental health conditions who have received specialized training to support the recovery of others with mental health conditions. This study examined the role of CPSs as educators of families and loved ones. Peer specialists joined established family support groups to provide education to family and loved ones. These educational sessions included sharing recovery stories and engaging in interactive question-and-answer sessions. Family members who participated in these sessions were invited to complete voluntary surveys designed to assess the impact of the CPS-led sessions. Forty-two participants responded across six sessions. On quantitative items, participants rated overall session quality as high and indicated that CPS-led sessions improved their understanding of the experience of mental health conditions, strengthened their belief in recovery, and increased their knowledge of how to support a loved one with a mental health condition. Thematic analysis of open-ended responses indicated that participants joined these sessions to gain a better understanding of CPS work, improve strategies to help loved ones, build community, and foster personal connections. More than half of participants reported gaining skills and resources from the program. This work adds to the literature supporting peer specialists in educational roles, including as educators of mental health providers across numerous disciplines. To our knowledge, this is the first report to specifically address the role of CPSs as educators of families, and additional research is needed in this area.

**Supplementary Information:**

The online version contains supplementary material available at https://doi.org/10.1007/s10597-026-01622-9.

##  Introduction

Certified peer specialists (CPSs) are care providers with lived experience of mental health conditions who have received specialized training enabling them to provide a broad range of services based on the recovery model (Cabral et al., 2014; Salzer et al., 2010). These services include direct peer support for people facing mental health experiences, education of staff, and advocacy for systemic changes to support the rights and dignity of people with mental health conditions. CPSs help to bridge the gap between patients and clinical mental health professionals with a unique perspective grounded in their personal recovery stories. Experienced CPSs also often develop a deep understanding of the mental healthcare system and the experiences of those who are navigating it through their own life experiences and their work within varying organizations. 

At McLean Hospital in Belmont, Massachusetts, USA, a private not-for-profit teaching hospital, CPSs engage in a broad range of teaching opportunities based on their expertise, including didactic teaching of staff and trainees, Grand Rounds, and supervision of trainees. The “peers as teachers” model for the education of mental health staff, psychiatry residents, and psychology fellows has received moderate attention in the literature (Millman et al., 2025). CPS education of mental health providers has been found to be an evidence-based strategy to improve providers’ understanding of recovery (Agrawal et al., 2016), promote empathy (Agrawal et al., 2016; Hayes et al., 2018; Valenti & Allred, 2020), reduce stigma (Mellsop et al., 2012), and improve communication with clients (Valenti & Allred, 2020). 

However, one under-studied aspect of peer-led education is the role of CPSs in teaching and supporting family members and invested parties of people with mental health conditions. To explore this, CPS members of our team, along with other CPSs at McLean Hospital, facilitated open-ended discussion sessions within existing family support groups across the hospital. These sessions, called “Peers as Teachers to Families,” were designed to offer families and loved ones hope, insight, logistical support, and strategies for navigating family interactions from the perspectives of CPSs. These sessions were similar in their goals to the National Alliance on Mental Illness “In Our Own Voice” (IOOV) series, though the structure of the “Peers as Teachers to Families” sessions varied and did not necessarily follow the IOOV format (Brennan & McGrew, 2013). Our sessions were also presented specifically with families and involved persons in mind, while IOOV is intended for both consumers and non-consumers. 

Building on reports of positive experiences from patients and families, we conducted a survey-based study to examine the specific impacts of CPS-led education on the families of individuals with mental health conditions. We hypothesized that CPS-delivered services would enhance families’ understanding of mental health conditions, strengthen their belief in recovery, and increase their awareness of how to support a loved one living with a mental health condition.

## Methods

### Participants

Survey respondents (*n* = 42) were primarily family members of individuals with mental health conditions. While most participants were parents, other relatives and involved individuals were also invited, as determined by each support group. Attendance was open, with no exclusion criteria, and participants were permitted to attend as many sessions as they wished. Given their connection to McLean Hospital clients, most respondents resided in Massachusetts and the broader New England region. The level of care in which family members or loved ones were engaged was not recorded. All participants had access to the technology required for virtual sessions.

### Session Structure

Peers as Teachers to Families sessions took place in established family support groups across three different outpatient clinical programs. The established groups that hosted the Peers as Teachers to Families sessions were either facilitated by non-CPS clinicians, or co-facilitated by CPSs and clinicians, and met on a weekly to monthly basis.

All Peers as Teachers to Families sessions were conducted virtually. Sessions lasted approximately one hour. Participants received advanced notice that a CPS would be facilitating the session. Each session had approximately 8–12 participants in attendance, and sessions were structured according to the preference of the CPS. Multiple sessions featured CPSs sharing their recovery stories in a 30–40-minute segment, followed by a question-and-answer (Q&A) period. In others, they began with a brief vignette of their recovery story and devoted more time to extended Q&A and open discussion. Over the course of two years (2021–2023), six recovery Peers as Teachers to Families sessions were held.

### Measures

#### Peers as Teachers to Families Survey

The study procedure consisted of voluntary surveys distributed to family support group participants at the end of each session. While intended as a one-time survey, no identifying information was collected and participants who attended multiple CPS-led sessions were not barred from completing the survey again. However, CPSs who led the sessions anecdotally noted that some participants declined to complete the survey because they had completed it previously. The Peers as Teachers to Families Survey was developed by McLean Hospital’s Peer Research Consortium, a multidisciplinary team of peers, clinicians, and researchers dedicated to advancing knowledge of peer work. Study participants completed an 11-question survey at the end of a peer-led session (see Online Material 1). The survey included open-response fields, multiple-choice questions, and 7-point Likert-type scales designed to assess the impact of the CPS-led session on participants. Surveys were administered in English and completed independently via Research Electronic Data Capture (REDCap). The study procedures were reviewed and a waiver was granted by the Mass General Brigham Institutional Review Board.

#### Demographics and Session Quality

Participants were asked whether they had prior knowledge of CPSs before attending each CPS-led session. Two items assessed which CPS-led services participants and their loved ones with mental health conditions had previously utilized. Overall quality of the session was rated on a 7-point scale, ranging from 1 (poor) to 7 (excellent).

#### Impact of CPS Services

Three items measured the perceived impact of each CPS-led session on participants’ outlook, using a 7-point scale from 1 (significantly decreased) to 7 (significantly increased). Specifically, participants were asked how the session influenced: (a) their understanding of the experience of living with a mental health condition; (b) their belief in the possibility of recovery; and (c) their knowledge of how to support a loved one with a mental health condition.

#### Motivations and Outcomes

Participants provided open-ended responses describing their motivations for initially attending the group as well as what they learned from the CPS-led session. They were also asked to rate the extent to which they believed family services provided by CPS differ from those provided by non-CPS, using a scale from 1 (does not differ at all) to 7 (is completely different). An optional free-response field allowed participants to elaborate on their ratings.

## Data Analysis

Quantitative responses were summarized using descriptive statistics. Thematic analysis was applied to the free-text responses, identifying key motivations and overarching impressions following the six-step framework developed by Braun and Clarke (2006). Codes were initially generated by a member of the research team using an inductive approach. These codes were then reviewed and revised by a second researcher until key themes were defined in the data set. Once complete, the final array of themes were shared with the broader Peer Research Consortium and finalized.

### Results

#### Demographics and Session Quality

**Fig. 1:**
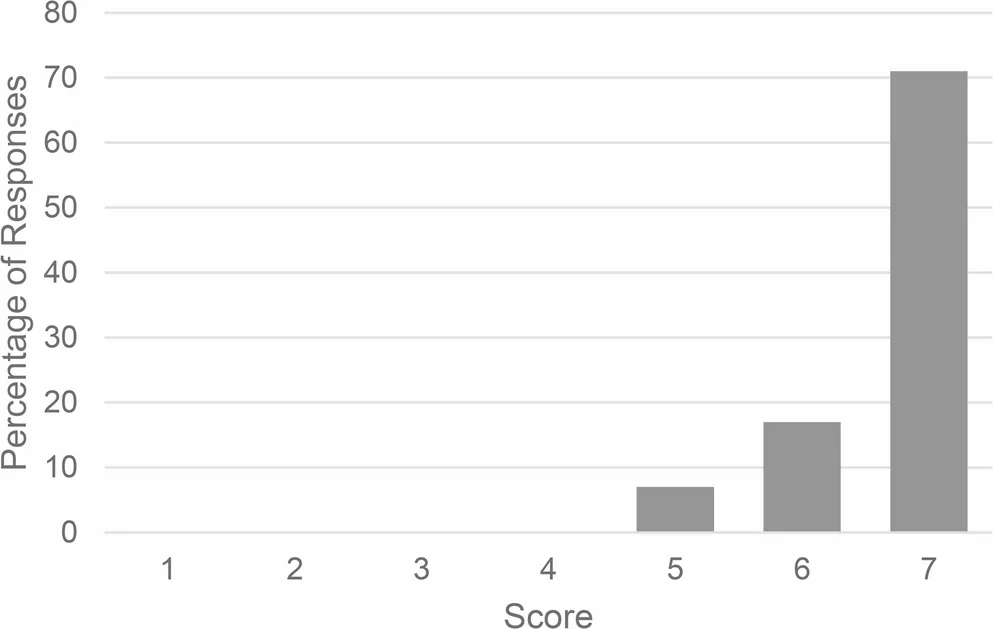
Overall quality of session on a scale from 1 (poor) to 7 (excellent)

The total sample consisted of 42 survey responses collected across six sessions. Because survey completion was voluntary, this number does not represent all session attendees, and the degree of missingness varied as no items were required. As no identifying information was collected, it is possible that some participants completed the survey more than once. Among respondents, 19% (n = 8) reported no prior knowledge of CPSs before attending the CPS-led session. The majority (71%, n = 30) indicated that they had previously engaged with one or more CPS-led groups within the hospital, and over half (52%, n = 22) reported that their loved ones had worked with a CPS at McLean Hospital. CPS engagement occurred across diverse settings, including inpatient units, outpatient clinics, community engagement events, community-based support programs, and family support groups. Overall session quality was rated highly (n = 40, M = 6.68, SD = 0.62; see Fig. 1).

#### Impact of CPS Services

**Fig. 2:**
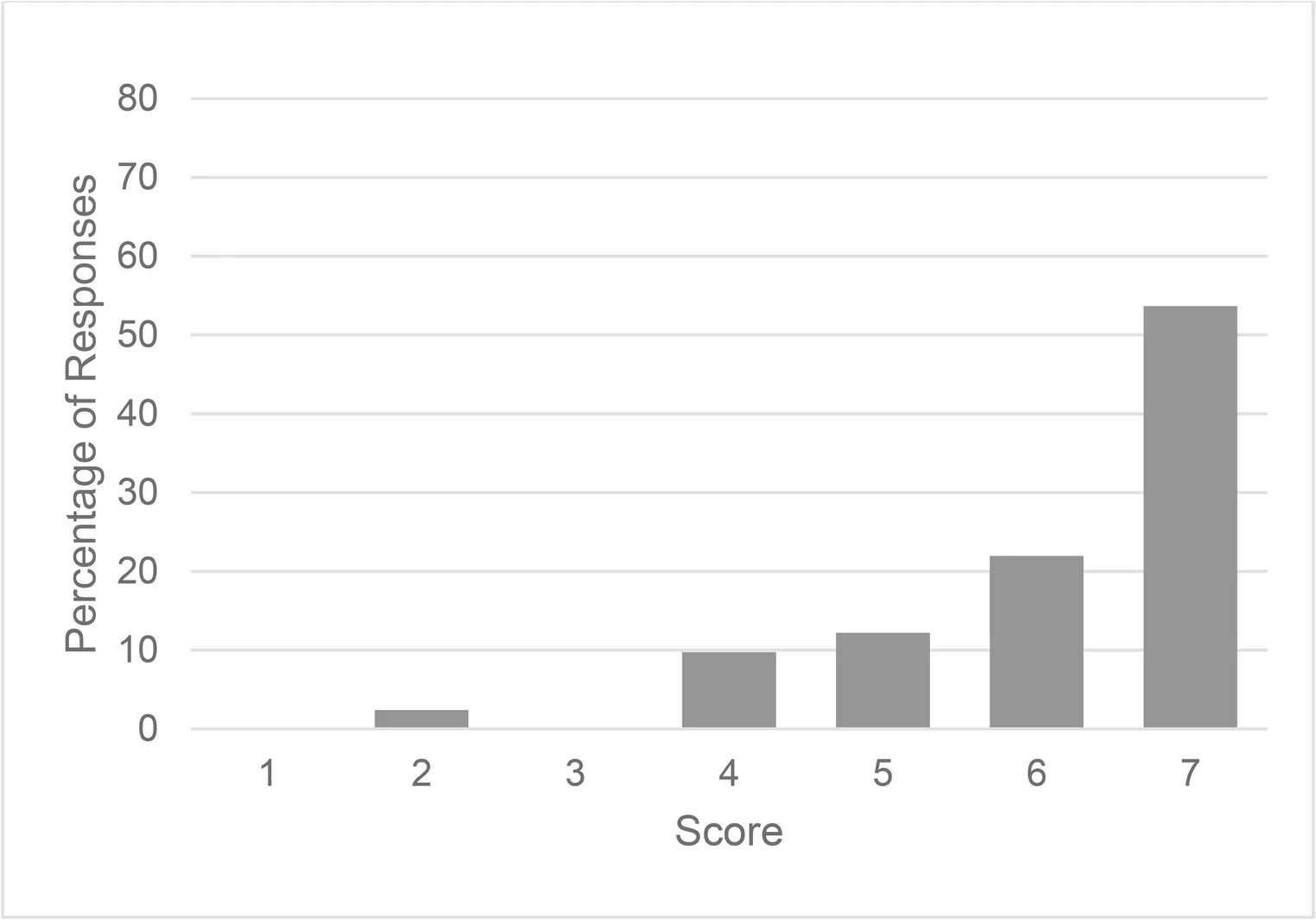
Impact on understanding of the experience of having a mental health condition on a scale from 1 (significantly decreased) to 7 (significantly increased)

**Fig. 3:**
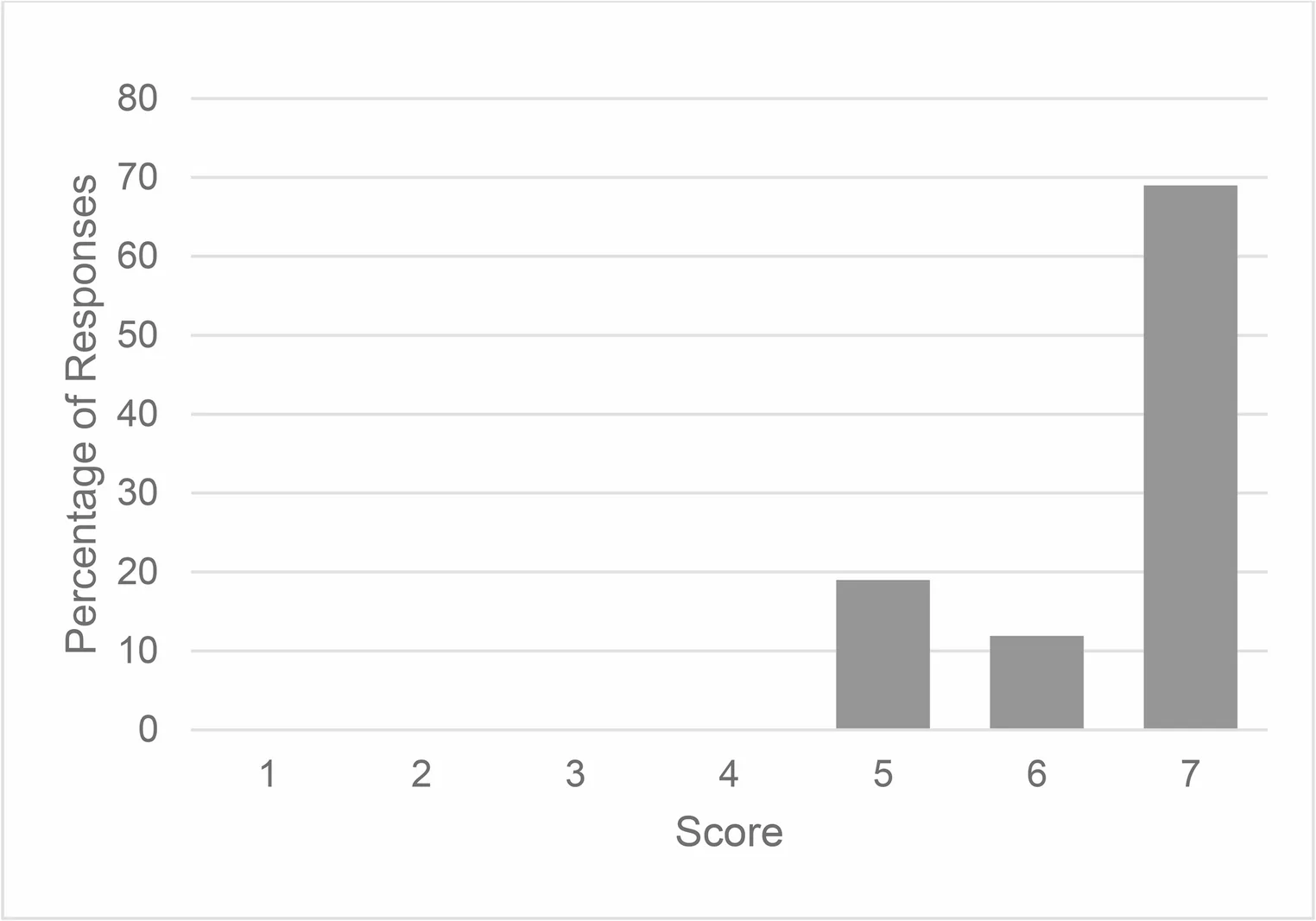
Impact on belief that people with mental health conditions can experience recovery, on a scale from 1 (significantly decreased) to 7 (significantly increased)

**Fig. 4:**
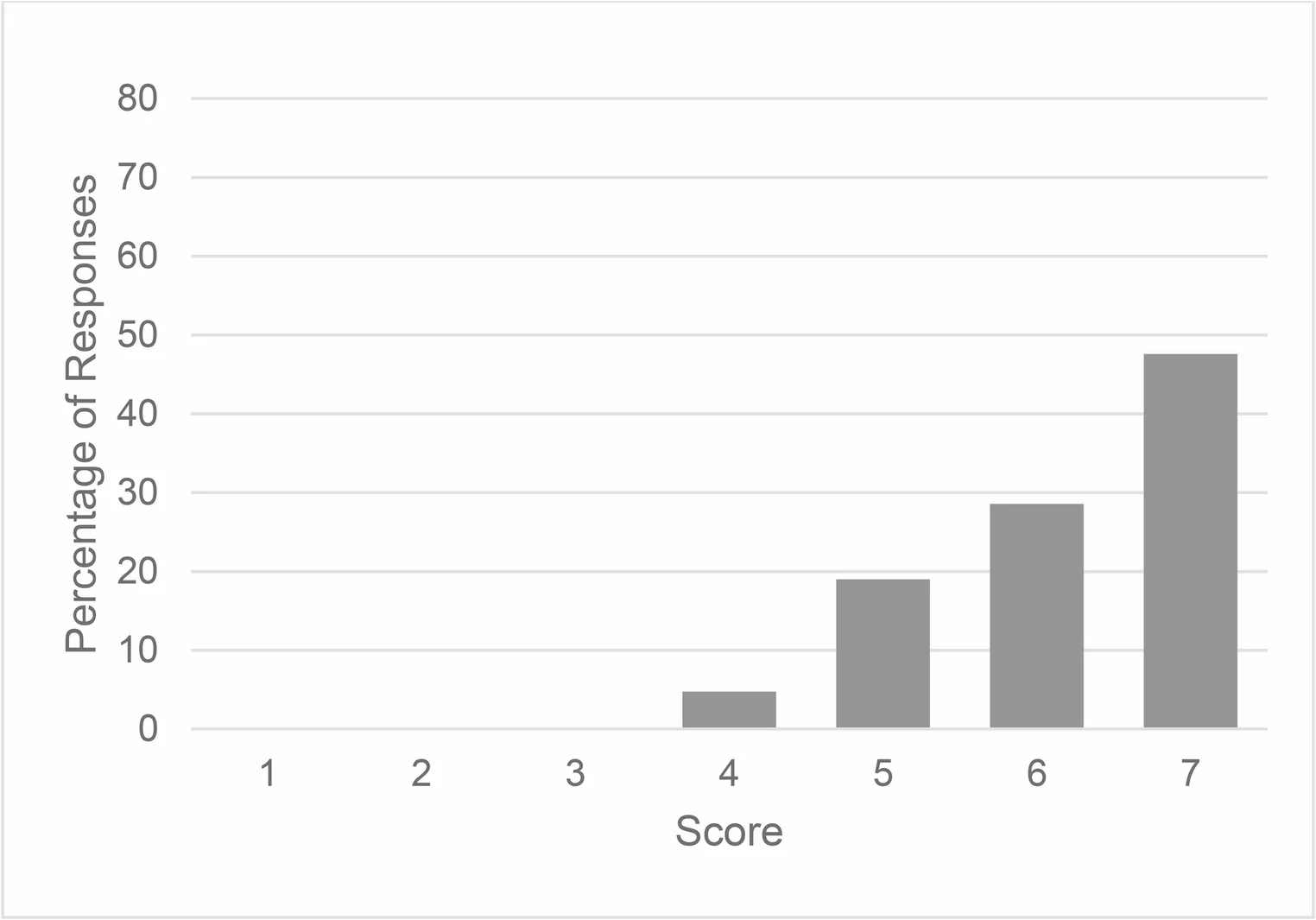
Impact on learning how to support a loved one with a mental health condition, on a scale from 1 (significantly decreased) to 7 (significantly increased)

Participants reported that the CPS-led sessions increased their understanding of the experience of having a mental health condition (*n* = 41, *M* = 6.12, *SD* = 1.21; see Fig. 2), strengthened their belief that people with mental health conditions can experience recovery (*M* = 6.5, *SD* = 0.80; see Fig. 3), and increased their knowledge of how to support a loved one with a mental health condition (*M* = 6.19, *SD* = 0.92; see Fig. 4).

#### Motivations and Outcomes

**Fig. 5:**
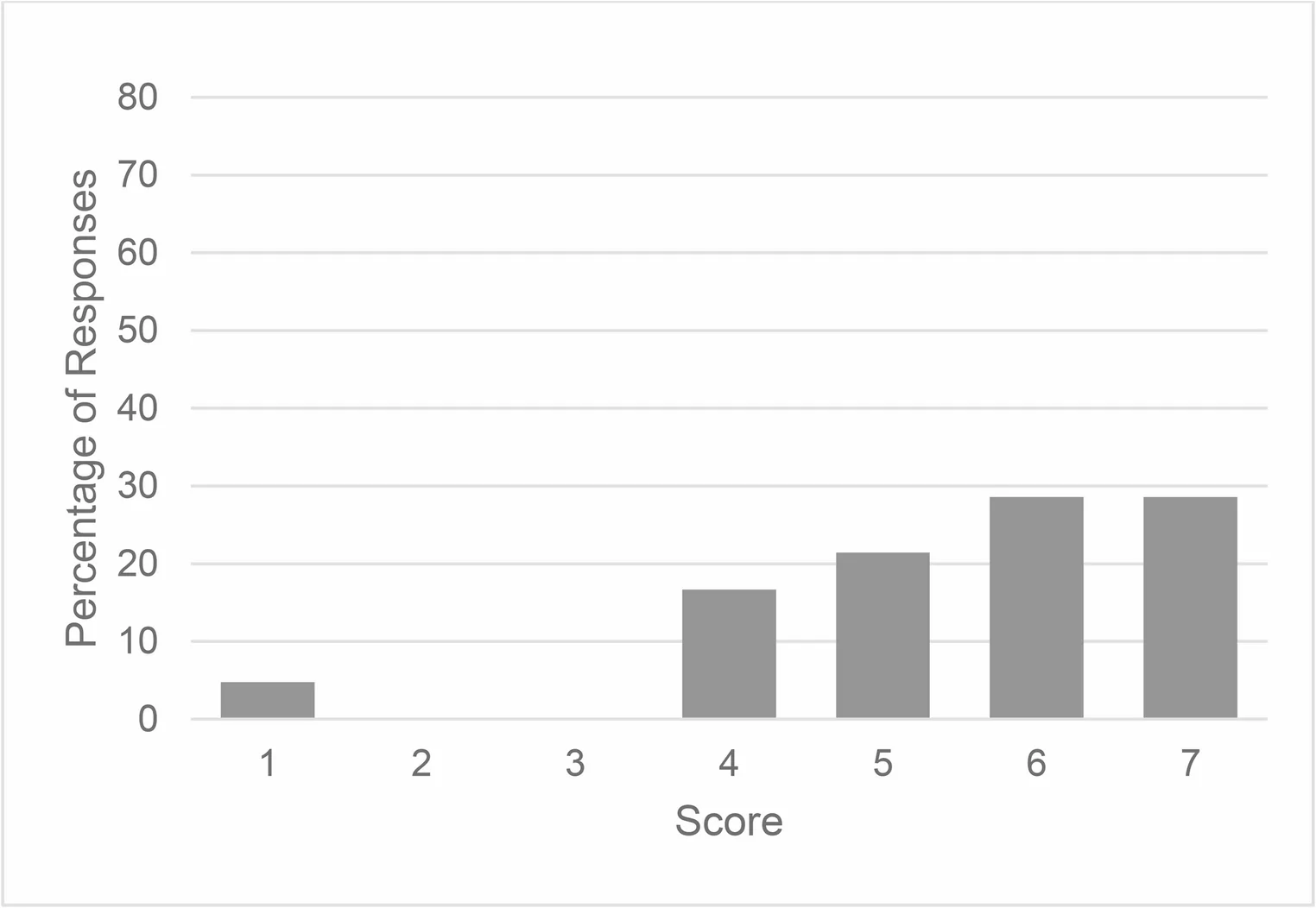
Perceived difference in services provided by peers versus non-peers, on a scale from 1 (does not differ at all ) to 7 (is completely different)

Most respondents rated services provided by CPSs as distinct from those provided by non-CPS (*M* = 5.5, *SD* = 1.47; see Fig. 5), with only two participants reporting no difference. A smaller proportion of participants viewed both perspectives as equally valuable (9%, *n* = 3), while another 9% (*n* = 3) remained undecided, citing limited exposure to CPS services (see Fig. 5).Open-ended responses further elaborated on the impressions of those who viewed peer services as distinct from non-peer services (*n* = 32), from which six themes were derived. The majority of comments emphasized the benefits of CPS work: 41% (*n* = 13) highlighted the unique and valuable perspective offered by lived experience; 22% (*n* = 7) noted that CPS possess greater credibility and knowledge than non-CPSs; 16% (*n* = 5) described CPS as offering deeper empathy and personal connection; and 3% (*n =* 1) indicated that CPSs provide hope for recovery (see Table 1).

**Table 1:** Most common reasons why participants felt CPS services were distinct from non-peer services (*n* = 32)

*n*	Theme
13	The lived experience of peer specialists provides a unique and valuable perspective
7	Peer specialists convey greater credibility and/or knowledgeability than non-peers
5	Peer specialists display greater empathy and personal connection than non-peers

#### Reasons for Attendance

Thematic analysis of reasons for attendance produced four themes and ten subthemes: CPS work, skill building, community, and personal connection (see Table 2). Half of the participants (*n* = 21) indicated that they attended for reasons related to the CPS speaker, including prior familiarity with the CPS (*n* = 2), finding hope for the future (*n* = 3), and specifically attending to hear the recovery story and perspective of a CPS (*n* = 16). Nearly half of all participants (*n* = 19) attended CPS-led sessions for skill-building purposes, including learning strategies to support a person of concern in recovery (*n* = 9), gaining deeper understanding of mental illness, interventions, and the recovery process (*n* = 6), and acquiring resources and knowledge (*n* = 4).

**Table 2:** Reasons why family members attended peers as teachers to families sessions

CPS Work	38%	To hear the recovery story & perspective of a peerspecialist
	7%	To find hope for the future
	5%	Prior familiarity with peer
Skill Building	21%	To learn strategies to support family member inrecovery
	14%	To gain deeper understanding of mental illness,interventions, and recovery process
	10%	To acquire resources and knowledge
Community	17%	To share issues and receive support fromcommunity
	14%	Regular attendee of support group
	2%	To help others
Personal Connections	19%	Family member in recovery

One third of participants (*n* = 14) attended for community-oriented reasons, including sharing issues and receiving support from CPSs (*n* = 7), helping others (*n* = 1), or were regularly attendants of the support group (*n* = 6). Finally, less than one third (*n* = 8) indicated that their attendance was motivated by personal connection to a loved one in recovery.

#### Lessons Learned

Thematic analysis of lessons learned produced three main themes and eleven sub-themes: CPS work, skill building, and community (see Table 3). Nearly half of all participants (*n* = 20) indicated that they learned the value of CPS as a resource (*n* = 4), expressed appreciation for the CPS speaker (*n* = 2), and/or gained hope for future recovery of their loved ones (*n* = 14).

**Table 3:** What family members learned from peers as teachers to families sessions

CPS Work	33%	Hope for future recovery
	19%	Value of peer specialists as a resource
	2%	Appreciation for peer specialist
Skill Building	21%	Strategies to support family member in recovery
	17%	Information regarding programs, methods to advocate, and additional resources
	14%	Importance of multimodal interventions (e.g. therapy, peer support, medications, etc.)
	14%	Increased awareness of hierarchical structures, stigmas, and language
	7%	Patience
	5%	Respecting autonomy and boundaries of family members in recovery
	2%	Understanding perspective of individuals with psychosis
Community	10%	Strength in community

More than half of all participants (*n* = 24) reported gaining a variety of skills and resources from the presentation. These included strategies to support a person of concern in recovery (*n* = 6), information regarding programs, methods to advocate, and additional resources (*n* = 4), the importance of multimodal interventions (*n* = 4), increased awareness of hierarchical structures, stigma, and language (*n* = 3), patience (*n* = 3), how to respect the autonomy and boundaries of a person in recovery (*n* = 2), and an understanding of the perspective of individuals with mental health conditions (*n* = 2). Three respondents indicated that they found strength in community.

## Discussion

CPS-led education is an under-studied facet of medical education generally (Agrawal et al., 2016), and it is particularly understudied as a method of family education in medical settings. Our aim in this study was not only to deliver CPS-led teaching sessions for the value they provide to families, but also to evaluate their educational impact. In this novel study, the role of CPSs as educators to family members and loved ones of people with mental health conditions was supported by both quantitative and qualitative data. Our findings suggest a strong interest among families in learning from CPSs, as well as distinct and meaningful benefits unique to this form of education.

In our study, nearly half of participants attended the sessions to build skills from CPS, seeking a deeper understanding of mental health conditions, strategies to support people in recovery, and access to resources and practical insights. Our four CPS teachers collectively brought 27 years of professional experience as CPSs providing care to clients and families, alongside many more years of lived experience with serious mental illness and navigating their own family dynamics. This combination of professional expertise and lived experience yields a depth of knowledge and wisdom that is qualitatively distinct from that obtained through formal medical education.

Participants in the Peers as Teachers to Families sessions consistently highlighted the unique value of CPS-led education. Nearly half of participants indicated that they learned the value of CPSs as a resource. More than half indicated that they gained concrete skills and tools to support people in recovery. Several participants expressed that CPSs have greater credibility and knowledge than non-CPSs. These findings underscore the depth of informal knowledge and nuanced insights that emerge from lived experience—an educational contribution that complements, but cannot be substituted by, non-peer services.

Our findings add to the current literature showing that peers are helpful in educational roles (Agrawal et al., 2016; Hayes et al., 2018; Mellsop et al., 2012; Valenti & Allred, 2020). In addition to community support and hope, we found that participants in the Peers as Teachers to Families sessions learned valuable skills and resources to support recovery. Just as mental health research is strengthened when individuals with lived experience of a condition are included throughout the research process (Hasking et al., 2025; Mjøsund et al., 2016), our findings support the idea that people with lived experience can contribute valuable lessons as educators. Our results support the feasibility of CPSs leading educational projects within academic institutions, conducting sessions independently, and serving as educators who bring unique insight and knowledge grounded in lived experience.

Certain aspects of our setting and population may limit the generalizability of our results. Our hospital employs numerous CPSs and values the CPS perspective. Not all institutions take a favorable view of peer work, and our study population may be skewed toward families of individuals who more closely align with our hospital’s philosophy. More work is needed across multiple institutions to better understand generalizability and how to support the greater population of CPSs in continuing to provide these services.

This was a quality improvement clinical project with highly focused data collection. To promote anonymity, we did not obtain potentially identifying information, thus one limitation of our results is the absence of demographic data and information about repeat attendance. While group leaders observed that some participants attended multiple sessions, it is unclear whether those individuals completed the survey more than once, which could have influenced our results. Additionally, we did not have a mechanism to compare the effectiveness of different CPS teachers or educational approaches across groups. In future studies, we plan to collect a larger sample and to include more detailed questions about participants’ experiences with peer teaching, including which aspects of the sessions were most helpful.

Our experience also suggests that perceived value of CPSs as teachers may increase with multiple sessions. In future projects, we hope to track participants who attended multiple sessions, thus permitting comparison of single- vs. multiple-session attendance. We also hope to assess differences between individual CPS teachers and differing teaching formats. Moreover, we plan to study the impact of peer teaching interventions on staff members and trainees, as early work suggests considerable benefits to this approach (Millman et al., 2025).

## Conclusions

Our findings contribute to the growing literature on CPS education, demonstrating that CPS-led teaching is highly effective for families and offers a knowledge base that is equally valuable—though distinct—from formal medical education. There is a critical need to empower peers to contribute as researchers, teachers, and authors, as much of the psychiatric literature, including work on CPSs, does not include their direct involvement. Notably, there has been very little work on CPS education for families in medical settings, suggesting that a significant body of knowledge and insight remains absent from the current psychiatric educational literature. Further research on CPS-led teaching, including education of family members, is essential to filling this gap. Our “Peers as Teachers to Families” intervention illustrates that when CPSs are empowered to teach family members and other involved individuals, CPS perspectives provide important insights, practical wisdom, and value that are distinct from those offered in other settings.

## Supplementary Information

Below is the link to the electronic supplementary material.

Supplementary Material 1

## Data Availability

Due to the small size of the groups surveyed and privacy of the support group format, the collected questionnaire results are not publicly available. Individuals with questions about the information collected are welcome to contact the study authors.
