# Supplementary material for: The Impact of Certified Peer Specialists as Teachers to Families
Source: Community Ment Health J. 2026 Apr 16;62(7):1292–9. doi: 10.1007/s10597-026-01622-9 (PMC13576794; doi:10.1007/s10597-026-01622-9)
Supplement: Supplementary file 1 [file 10597_2026_1622_MOESM1_ESM.docx]

**Supplementary Material**

**Online Resource 1**

*Peers as Teachers to Families Survey*

| 1. Please briefly share why you attended today's group: | *(open response)* |
| --- | --- |
| 2. Did you know about peer specialists prior to today's group? | ○ Yes  ○ No |
| 3. Please rate the overall quality of this session on a scale from 1 (poor) to 7 (excellent). | ○ 1  ○ 2  ○ 3  ○ 4  ○ 5  ○ 6  ○ 7 |
| 4. Please rate the impact this peer specialist session has had on your understanding of the experience of having a mental health condition 1 (significantly decreased) - 7 (significantly increased). | ○ 1  ○ 2  ○ 3  ○ 4  ○ 5  ○ 6  ○ 7 |
| 5. Please rate the impact this peer specialist session has had on your belief that people with mental health conditions can experience recovery (i.e. lead a personally fulfilling life). 1 (significantly decreased) - 7 (significantly increased). | ○ 1  ○ 2  ○ 3  ○ 4  ○ 5  ○ 6  ○ 7 |
| 6. Please rate the impact of this peer specialist session has had on learning how to support a loved one with a mental health condition. 1 (significantly decreased)-7 (significantly increased). | ○ 1  ○ 2  ○ 3  ○ 4  ○ 5  ○ 6  ○ 7 |
| 7. In your view, do services for families that are provided by peers differ from services for families that are provided by non-peers? 1 (does not differ at all) - 7 (is completely different) | ○ 1  ○ 2  ○ 3  ○ 4  ○ 5  ○ 6  ○ 7 |
| 8. Optional free response: Please explain your rating for question 7. | *(open response)* |
| 9. In what capacity have you worked with a peer specialist at McLean? Check all that apply. | ❒ PACT team  ❒ Wellspace  ❒ AB2  ❒ NB2  ❒ Waverly Place  ❒ OnTrack family group  ❒ Sharing Recovery Night  ❒ AB2 family support group |
| 10. In what capacity has your loved one with a mental health condition worked with a peer specialist at McLean? | ❒ PACT team  ❒ Wellspace  ❒ AB2  ❒ NB2  ❒ Waverly Place  ❒ OnTrack family group  ❒ Sharing Recovery Night  ❒ AB2 family support group  ❒ Not sure  ❒ None |
| 11. What did you learn from today's group? | *(open response)* |
